# Outcomes of arthroscopic debridement of isolated Ligamentum Teres tears using the iHOT-33

**DOI:** 10.1186/s12891-017-1905-6

**Published:** 2017-12-29

**Authors:** Nicholas Pergaminelis, Jesse Renouf, Camdon Fary, Oren Tirosh, Phong Tran

**Affiliations:** 1 Orthopaedic Department, Western Health, Footscray, VIC 3011 Australia; 2 The University of Melbourne, Parkville, VIC Australia; 3 Australian Institute for Musculoskeletal Science (The University of Melbourne and Western Health), St. Albans, VIC Australia

**Keywords:** Hip arthroscopy, Ligamentum Teres, Outcomes, International hip outcome tool

## Abstract

**Background:**

Tears of the Ligamentum Teres are a common cause of groin pain. Tears of the ligament frequently co-exist alongside other bony or labral intra-articular hip lesions, which are also treated at the time of arthroscopy. For this reason, it is often difficult to assess the effect that debridement alone has on improving patient pain and function. This study aims to assess the short-term outcomes of arthroscopic debridement of isolated Ligamentum Teres tears using a validated patient-reported outcome score – the International Hip Outcome Tool (iHOT-33).

**Methods:**

A retrospective review was performed of 35 patients (37 hips) who had an isolated Ligamentum Teres tear treated with arthroscopic radiofrequency debridement between September 2012 and September 2015. Patients with concomitant intra-articular hip pathology (femoroacetabular impingement, labral lesions, hip dysplasia) were excluded from the study. All patients completed the iHOT-33 questionnaire pre-operatively and post-operatively. The mean age was 37.7 years (range 16–67 years) and patients were followed up for a mean period of 17.7 months (range 6–42 months). Thirty-one patients were female and 4 were male.

**Results:**

Thirty-seven isolated partial tears were managed with arthroscopic radiofrequency debridement. At follow up, the mean iHOT-33 score improved from 26.9 pre-operatively to 48.0 post-operatively, representing a mean improvement of 21.1 (*p* < 0.001). The minimum clinically important difference of the iHOT-33 is 6.1 points. Significant improvements were noted in all four sub-sections of the iHOT-33.

**Conclusion:**

Arthroscopic radiofrequency debridement of isolated Ligamentum Teres tears provides short-term benefit in the majority of patients, including significant improvement in sporting function.

## Background

Ligamentum Teres tears are being increasingly diagnosed during hip arthroscopy [1–3]. Having long been considered a vestigial structure of little significance, tears of the ligament are now recognised as a common cause of debilitating hip pain and mechanical hip symptoms [2–4]. They occur most frequently in young, active females, and are today routinely managed during hip arthroscopy with radiofrequency debridement [3, 4]. However, tears of the Ligamentum Teres are often diagnosed and managed arthroscopically at the same time as other co-existing bony or labral intra-articular hip pathology. It has therefore proved difficult to assess the effect that arthroscopic debridement of the ligament alone has on patient symptoms. Indeed, studies which solely evaluate the outcomes of arthroscopic debridement of isolated Ligamentum Teres tears remain limited [3, 4]. Previous studies have produced encouraging preliminary results but none have thus far utilised the International Hip Outcome Tool (iHOT-33) – a patient-reported outcome questionnaire that has been validated and recommended for use in hip arthroscopy and hip preservation surgery [5]. The aim of this study was to assess the short-term outcomes of arthroscopic debridement of isolated tears of the Ligamentum Teres using the iHOT-33.

## Methods

A retrospective review of a hip arthroscopy database was carried out. The database included 2541 hip arthroscopies performed by two surgeons experienced in the procedure between September 2012 and September 2015. Of these, 557 (22.7%) involved debridement of a tear of the Ligamentum Teres. Patients who had concomitant intra-articular hip pathology (femoroacetabular impingement, labral lesions, hip dysplasia) also managed during arthroscopy were excluded from the study, as were patients who had previous hip surgery.

The study ultimately included 37 operations in 35 patients who had had an isolated Ligamentum Teres tear managed with arthroscopic debridement. There were two indications for surgery in these patients. 1) Evidence of an isolated Ligamentum Teres tear on pre-operative MRI. 2) Undifferentiated hip/groin pain refractory to conservative management, with no intra-articular hip pathology identified on pre-operative imaging. The patient population had a mean age of 37.7 years (range 16–67 years) and were followed up for a mean period of 17.7 months (median 16.6 months, range 6–42 months). The majority of the patients were female (31 females, 4 males). Twelve arthroscopies were performed on the left hip and 25 on the right hip. Patients were most commonly involved in amateur netball, gymnastics, dancing and running (Table 1).

**Table 1:** Patient demographics

	Number
Number of patients	35
Mean age	37.7
Female: Male	31: 4
Number of hips	37
Left: Right	12: 25
Sport / Activity	
Netball	6
Gymnastics	2
Dancing	2
Running	2
Horse-riding	1
Rowing	1
Soccer	1
Basketball	1
Cycling	1
Swimming	1

### Surgical technique

All arthroscopies were carried out by two surgeons experienced in the procedure. Surgeries were performed in the lateral decubitus position under general anaesthesia (without muscle relaxants), in a manner consistent with the method described by Glick [6]. Traction was applied to the operative leg using a McCarthy Hip Distractor (Innomed, Inc., Savannah, GA, USA), and two portals (direct lateral and anterolateral) were established using an image intensifier. Distension of the joint was maintained during the procedure using an arthroscopic pump, and pressure was kept constant at 40 mmHg. A 70^0^ arthroscope was used throughout and an initial assessment of intra-articular pathology was performed. The Ligamentum Teres was routinely assessed in external rotation and internal rotation. Where tears of the Ligamentum Teres were present, they were classified as partial or complete in accordance with Gray and Villar’s classification system [7]. The presence of associated chondropathy was also noted, and was graded according to the Outerbridge classification system [8]. In the setting of chondropathy, mild chondroplasty was performed in some cases. All tears of the Ligamentum Teres were debrided using a radiofrequency ablation probe (Vulcan Eflex Ablator Probe, Smith & Nephew, Andover, MA, USA). Where synovitis was present, a localised or generalised synovectomy was also performed, also using a radiofrequency ablation probe. Post-arthroscopy, the joint was injected with local anaesthetic (Ropivicaine 150 mg) and morphine (5 mg). All surgeries were performed as day-cases or overnight stays. Post-operatively, crutches were used as required in the days following surgery and patients were advised to fully weight-bear. A standard rehabilitation protocol was adhered to in all cases [9].

### Outcome score

Patients completed the iHOT-33 pre-operatively and post-operatively. The iHOT-33 is a self-administered patient-reported outcome questionnaire that was developed in 2012 for active patients between the ages of 18 and 60 with a variety of hip pathologies [10]. It has been recommended as the most appropriate tool for evaluating the outcomes of hip arthroscopy in young, active patients [11, 12]. The iHOT-33 contains 33 questions and is divided into four sub-sections, each of which assesses a distinct domain of a patient’s daily life. It culminates in a total score from 0 to 100, where 100 represents an optimal score. Both the total iHOT-33 scores and the average scores for the questionnaire’s four sub-sections were analysed.

### Statistical analysis

Mean pre-operative and post-operative iHOT-33 scores were calculated. The difference between the mean pre-operative and mean post-operative iHOT-33 scores were investigated with a paired sample t Test. P was be considered statistically significant if it was less than 0.05. Single-factor ANOVA was used to determine the effect of performing a synovectomy on patient outcomes.

## Results

Of the 37 hips operated on, partial tears of the Ligamentum Teres were identified in all cases. No patients had complete tears of the Ligamentum Teres. All patients presented with hip or groin pain, and 7 reported additional mechanical symptoms such as catching, clicking or giving way. In 12 cases patients could recall an acute, traumatic event that precipitated their symptoms, while they could not in 25 cases. On examination, all patients were found to have hip irritability, with 29 of these recording a positive Flexion Adduction Internal Rotation (FADIR) Test and 3 recording a positive Flexion Abduction External Rotation (FABER) Test.

Before orthopaedic referral and review, all patients had been managed conservatively by their general practitioner or a sports physician with analgesia and in some cases physical therapy. The duration of symptoms before specialist referral was highly variable, with a median period of 16 months (range 3 months – 5 years). Following their initial orthopaedic review, all patients chose to pursue arthroscopic intervention. The mean waiting time between orthopaedic review and surgery was 13 weeks (95% CI 9–17 weeks), and during this period patients continued conservative management for their symptoms.

All patients had a degree of synovitis detected at arthroscopy. A localised synovectomy was performed in 19 cases and a generalised synovectomy was performed in 9 cases (Table 2). Performing a synovectomy did not significantly impact post-operative iHOT-33 scores (*p* = 0.72). Associated chondral damage was present in 22 hips (Table 2). Eleven had both acetabular and femoral chondral damage, 8 had femoral chondral damage alone and 3 had acetabular chondral damage alone. Six patients underwent mild chondroplasty in for chondral damage. Capsular tightening was performed in addition to arthroscopic debridement of the Ligamentum Teres in 2 operations, once by radiofrequency debridement and once by suture plication. One patient had a second operation to debride their Ligamentum Teres due to persistent symptoms following their initial arthroscopy.

**Table 2:** Operative findings and details

	Number	Percent
Synovectomy		
Localised	19	51.4
Generalised	9	24.3
Chondropathy (Outerbridge Classification)		
*Femoral*	*18*	*48.6*
Grade 1	2	5.4
Grade 2	8	21.6
Grade 3	8	21.6
Grade 4	0	0
*Acetabular*	*12*	*32.4*
Grade 1	3	8.1
Grade 2	4	10.8
Grade 3	1	2.7
Grade 4	4	10.8
Capsular Tightening	2	5.4

At follow up, the mean total iHOT-33 score improved from 26.9 pre-operatively (95% CI 22.1–31.7) to 48.0 post-operatively (95% CI 40.6–55.3), representing a mean improvement of 21.1 (95% CI 14.2–27.9, *p* < 0.001). The minimum clinically important difference of the iHOT-33 is 6.1 points [10]. Significant improvements were noted in all four sub-sections of the iHOT-33 (Table 3). For the single patient who underwent a second arthroscopy, revision surgery led to an improvement of 26.7 points. Tears of the Ligamentum Teres were successfully diagnosed pre-operatively on MRI in only 9 cases (21.6%).

**Table 3:** Pre-operative and post-operative iHOT-33 scores

	Mean Pre-Operative Score	Mean Post-Operative Score	Mean Change	*p*
Section 1: Symptoms & Functional Limitations	33.6	57.2	23.6	< 0.001
Section 2: Sports & Recreational Activities	15.0	31.9	16.9	< 0.001
Section 3: Job-Related Concerns	38.4	56.1	17.7	0.007
Section 4: Social, Emotional & Lifestyle Concerns	19.1	42.6	23.6	< 0.001
Total Score	26.9	48	21.1	< 0.001

## Discussion

Tears of Ligamentum Teres are being increasingly identified at the time of arthroscopy [13]. There is also a growing body of evidence regarding the Ligamentum Teres’ role as a secondary stabiliser of the hip joint, as well as its potential nociceptive and proprioceptive function [14–16]. Debriding tears of the ligament (without resecting the ligament itself) potentially reduces inflammation around its torn fibres, which in turn relieves pain and improves its mechanical function.

This study demonstrated that arthroscopic debridement of isolated tears of the ligament leads to significant short-term improvements in both pain and function. After a mean follow up of 17.7 months, the mean iHOT-33 score improved from 26.9 pre-operatively to 48.0 post-operatively. These findings are consistent with positive results described elsewhere in the literature.

Byrd and Jones produced the first prospective case series that assessed the outcomes of arthroscopic debridement in 2004 [2]. Twenty-three patients (mean age 28.3) were followed for a mean period of 29.2 months. They noted a significant improvement in the mean Modified Harris Hip Score (MHHS) from 47 pre-operatively to 90 post-operatively. However, patients with concomitant intra-articular hip pathology were not excluded from their study.

Subsequently, two retrospective case series have evaluated the outcomes of arthroscopic debridement of the isolated Ligamentum Teres tear. Haviv and O’Donnell followed 29 patients (mean age 25) for a mean period of 30 months [3]. They observed significant improvements in both the mean MHHS (70 pre-operatively to 86 post-operatively) and Non-Arthritic Hip Score (NAHS) (65–86).

Similar improvements were noted in Amenabar and O’Donnell’s 2013 study of 26 patients (mean age 24.4), who underwent both arthroscopic debridement and capsular tightening by either radiofrequency or suture plication [4]. At follow up (mean 32 months), significant improvements in both the MHHS (65–89) and NAHS (66–87) were noted.

Numerous patient-reported outcome scores have been utilised to assess hip arthroscopy outcomes [5]. The Modified Harris Hip Score is derived from the Harris Hip Score, which itself was created for older, arthritic patients undergoing total hip arthroplasty [17]. As such, it has been shown to have a ceiling effect in younger patients [12]. Furthermore, it has been demonstrated to have limited correlation with patient satisfaction in the setting of hip arthroscopy [18]. The Non-Arthritic Hip Score was developed in 2003 for younger patients between the ages of 20 and 40 with non-arthritic hip pathology. It has good reliability and validity but is not validated for monitoring post-operative changes [19]. It has also been shown to have a ceiling effect in younger patients [12].

The iHOT-33 is validated for use in hip arthroscopy, particularly in young, active patients. Although it has been found to be a valid tool to measure hip arthroscopy outcomes, few publications have utilised it to date [20, 21]. The iHOT-33 was formulated in 2012 for active patients between the ages of 18 and 60 with a variety of hip pathologies [10]. It has excellent validity, and has been recommended by two recent systematic reviews as the most appropriate questionnaire in appraising outcomes of arthroscopic hip surgery [11, 12].

In addition, the iHOT-33’s four sub-sections further allow for further sub-analysis of surgical outcomes. Significant improvements were observed in all four sections of the iHOT-33 with the greatest improvements in the domains of ‘Symptoms and Functional Limitations’ and ‘Social, Emotional and Lifestyle Concerns’. Lesser levels of improvement were observed in the domains of ‘Job-Related Concerns’ and ‘Sports and Recreational Activities’.

Participation in sport is common amongst patients with tears of the Ligamentum Teres [2–4]. This study’s population were young and athletic, with patients most commonly participating in netball, gymnastics, dancing and running. As such, the findings related to the ‘Sports and Recreational Activities’ sub-section provide useful insights. The sub-sections of the iHOT-33 have not been validated for individual use, but it has been suggested that individual analysis may in fact provide a more valuable assessment of a patient’s functional limitations than their total iHOT-33 score [10, 22].

Clinical diagnosis of tears of the Ligamentum Teres is difficult, as findings on both history and examination are often non-specific for intra-articular hip pathology [2, 3, 23]. In this study, patients most commonly presented with hip or groin pain, with some experiencing mechanical symptoms such as clicking, catching and feelings of instability. A majority of patients could not recall an acute traumatic injury which preceded the onset of their symptoms. The natural history of these symptoms was heterogeneous, with patients being managed conservatively by general practitioners or sports physicians from 3 months to up to 5 years before being referred to an orthopaedic surgeon. Hip irritability and a positive FADIR sign were the most common findings on examination, reinforcing the non-specific nature of clinical findings. Like other intra-articular hip pathology, MRI is the preferred method of detecting tears pre-operatively, but multiple studies have found that it is neither sensitive nor specific [1, 24, 25]. Our study demonstrated improved detection rates. 21.6% of tears were successfully diagnosed pre-operatively on MRI, albeit in a relatively small population. This perhaps suggests that the ability to diagnose tears is improving as recognition of Ligamentum Teres pathology continues to increase. Nonetheless, MRI was still an unreliable diagnostic tool. Ultimately, all patients progressed to arthroscopy following their initial orthopaedic review, regardless of the nature of their symptoms, acuity of their presentation and initial radiological findings.

Although the use of the iHOT-33 and the exclusion of concomitant intra-articular hip pathology remain valuable aspects of this study, several limitations still exist. The most significant is that the study did not include a control group. Despite the fact that all patients had been unsuccessfully managed non-operatively before arthroscopic intervention, both the timeframe and nature of conservative management was highly variable. In addition, data collection was retrospective and there are inherent limitations of such a study design. While most of the study’s participants were young females, there was still a relative level of heterogeneity in their age and level of physical activity. Furthermore, follow up only occurred over a short-term period. Nevertheless, initial results are promising and represent the largest population of patients in the current literature. Extended follow up is now required, and is currently being performed, to assess the long-term outcomes of arthroscopic debridement.

## Conclusion

Arthroscopic debridement of isolated tears of the Ligamentum Teres leads to short-term improvements in pain and function, when using the iHOT-33 to measure outcomes. Improvements were most significant in the domains of symptoms and functional limitations, as well as social, emotional and lifestyle concerns. They were less marked in job-related and sporting activities. Despite this, significant improvement was observed in sporting function, providing positive and valuable prognostic information for the athletic patients who commonly suffer from tears of the ligament.

## Abbreviations

FABER: Flexion abduction external rotation test
FADIR: Flexion adduction internal rotation test
iHOT-33: International hip outcome tool
